# Solitary fibrous tumor of the pancreas

**DOI:** 10.4322/acr.2021.245

**Published:** 2021-03-12

**Authors:** Angela De La Hoz Rodríguez, Marcello Di Martino, Mariel Valdivia Mazeyra, Elena Martín-Pérez

**Affiliations:** 1 Hospital Universitario de la Princesa, General Surgery Department. Madrid, Spain; 2 Hospital Universitario de la Princesa, Pathology Department. Madrid, Spain

**Keywords:** General Surgery, Pancreas, Solitary Fibrous Tumors

Solitary fibrous tumors (SFT) are mesenchymal tumors with a haemangiopericytoma-like branching vascular pattern, which usually presents as a well-circumscribed mass. The majority of these tumors have been reported in the pleura.^1^ The pancreas is an unusual location for this tumor, with less than 20 cases described in the literature. The differential diagnosis of pancreatic SFTs includes several spindle cell neoplasms such as GIST, leiomyosarcoma, schwannoma, and fibromyxoid sarcoma.^2^ However, the definitive diagnosis of SFT can only be provided by the histological examination with the immunohistochemical analysis. Particularly, the growth pattern and the positivity for STAT-6 and CD 34 are helpful in differentiating SFTs from other mesenchymal tumors.^3^ There are scarce data about the biological behavior of SFT. Malignancy criteria include tumor size (>10 cm), infiltrative margins, high cellularity, nuclear pleomorphism, tumor necrosis and increased mitotic index (≥ 10 mitotic figures per 10 high powered fields).^4^ Therefore, complete surgical excision is the gold standard treatment and close follow-up is highly recommended since the prognosis of this tumor is uncertain.

Figure 1 refers to the imaging documentation of a 48-year-old woman with a history of orbital exenteration and maxillectomy for a synovial sarcoma ten years before. She was referred to the Hepatobiliary Surgical Clinic with mild abdominal pain and an abdominal mass in physical examination. The abdominal computed tomography scan (CT) showed a well-defined hypervascular pancreatic mass of 13 x 10 x 9.5 cm without evidence of distant metastasis. The mass caused portal vein (PV) displacement in a 6 cm segment and dilatation of the main pancreatic duct in the absence of pancreatic tail parenchyma atrophy (Figure 1A). Surgical intervention was scheduled. Total pancreatectomy with PV resection and end-to-end reconstruction with a left renal vein graft was performed (Figure 1B). The pathological analysis revealed a solitary fibrous tumor (SFT) with fusiform fibroblastic cells, scarce cytoplasm and no nuclear atypia. The lesion presented a hemangiopericytoma-like branching vascular pattern, with stromal collagen fibers surrounded by blood vessels with a “deer horn” stromal pattern (Figure 1C). Immunohistochemically, the tumor cells were positive for STAT-6: a sensitive and specific marker for solitary fibrous tumor (Figure 1D). All lymph nodes received in the specimen were negative. The postoperative course was uneventful, and the patient was discharged on the 12^th^ postoperative day. Chemo - radiotherapy was not necessary. No tumor recurrence was found in one year of follow-up.

**Figure 1:**
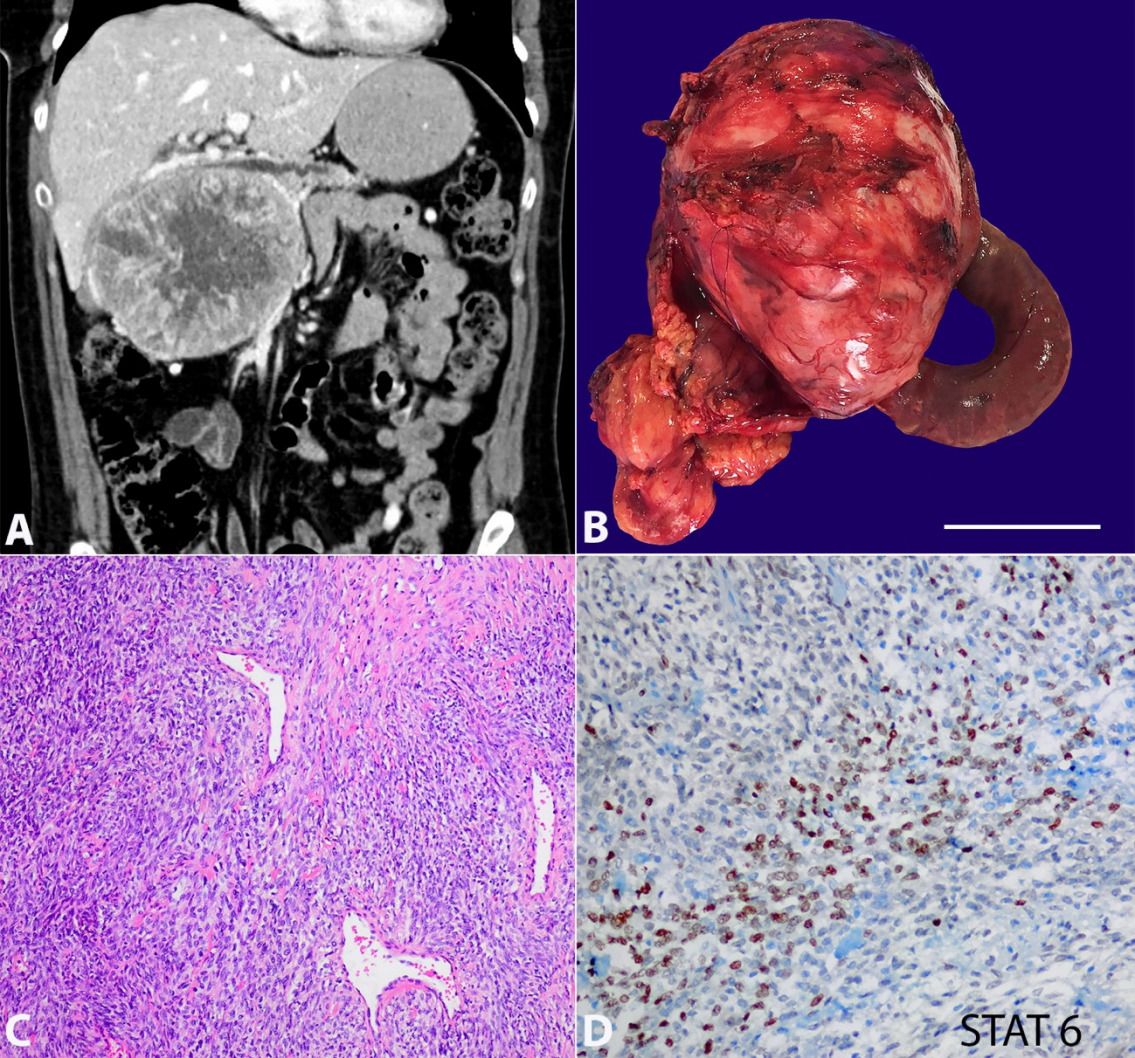
A – Abdominal computed tomography showing a well-circumscribed 13 x 10 x 9.5 cm mass in the head of the pancreas, portal vein displacement, and pancreatic main duct dilatation; B – Surgical specimen of total pancreatectomy and external surface of the tumor (scale bar: 7 cm); C – Hemangiopericytoma-like branching vascular pattern, with stromal collagen fibers surrounded by blood vessels with a “deer horn” stromal pattern (H&E, 10x); D – Immunostaining for STAT-6.

## References

[1] Han HS, Baek HY, Han SY (2015). Solitary fibrous tumor of the pancreas: A case report and review of the literature. Korean J Med.

[2] Spasevska L, Janevska V, Janevski V, Noveska B, Zhivadinovik J (2016). Solitary fibrous tumor of the pancreas: a case report and review of the literature. Pril.

[3] Paramythiotis D, Kofina K, Bangeas P, Tsiompanou F, Karayannopoulou G, Basdanis G (2016). Solitary fibrous tumor of the pancreas: case report and review of the literature. World J Gastrointest Surg.

[4] Gold JS, Antonescu CR, Hajdu C (2002). Clinicopathologic correlates of solitary fibrous tumors. Cancer.

